# Salinomycin inhibits cholangiocarcinoma growth by inhibition of autophagic flux

**DOI:** 10.18632/oncotarget.23339

**Published:** 2017-12-16

**Authors:** Johannes Klose, Engin Guerlevik, Tina Trostel, Florian Kühnel, Thomas Schmidt, Martin Schneider, Alexis Ulrich

**Affiliations:** ^1^ Department of General, Visceral and Transplantation Surgery, University of Heidelberg, Heidelberg 69120, Germany; ^2^ Department of Gastroenterology, Hepatology and Endocrinology, Hannover Medical School, Hannover 30625, Germany

**Keywords:** Salinomycin, cholangiocarcinoma, in vivo model, autophagic flux

## Abstract

**Introduction:**

Cholangiocarcinoma is characterized by aggressive tumor growth, high recurrence rates, and resistance against common chemotherapeutical regimes. The polyether-antibiotic Salinomycin is a promising drug in cancer therapy because of its ability to overcome apoptosis resistance of cancer cells and its selectivity against cancer stem cells. Here, we investigated the effectiveness of Salinomycin against cholangiocarcinoma *in vivo*, and analyzed interference of Salinomycin with autophagic flux in human cholangiocarcinoma cells.

**Results:**

Salinomycin reduces tumor cell viability, proliferation, migration, invasion, and induced apoptosis *in vitro*. Subcutaneous and intrahepatic cholangiocarcinoma growth *in vivo* was inhibited upon Salinomycin treatment. Analysis of autophagy reveals inhibition of autophagic activity. This was accompanied by accumulation of mitochondrial mass and increased generation of reactive oxygen species.

**Conclusions:**

This study demonstrates the effectiveness of Salinomycin against cholangiocarcinoma *in vivo*. Inhibition of autophagic flux represents an underlying molecular mechanism of Salinomycin against cholangiocarcinoma.

**Methods:**

The two murine cholangiocarcinoma cell lines p246 and p254 were used to analyze tumor cell proliferation, viability, migration, invasion, and apoptosis *in vitro*. For *in vivo* studies, murine cholangiocarcinoma cells were injected into syngeneic C57-BL/6-mice to initiate subcutaneous cholangiocarcinoma growth. Intrahepatic tumor growth was induced by electroporation of oncogenic transposon-plasmids into the left liver lobe. For mechanistic studies in human cells, TFK-1 and EGI-1 were used, and activation of autophagy was analyzed after exposure to Salinomycin.

## INTRODUCTION

Cholangiocarcinoma (CC) is one of the few remaining tumor entities that is characterized by very limited treatment options [1]. Radical surgical resection is the only chance to cure locally restricted disease. Of note, radical resection might be accompanied by mild or severe postoperative complications, such as small for size syndrome and consecutive liver failure after major liver resection [2–4]. However, most of the patients present with advanced or even metastatic stages of disease and are referred to palliative chemotherapy, which is rather ineffective. Only the combination of Gemcitabine and Cisplatin exerts some growth-inhibiting effects on CC in advanced stages of disease [5, 6]. Given that CC is the second most common primary liver cancer worldwide, new treatment options are of utmost importance.

Salinomycin (Sal) is a polyether antibiotic that exerts antitumoral effects on cancer stem cells *in vitro* and *in vivo* [7]. The antitumor effects of Sal were first described in breast cancer stem cells [8] and were confirmed in many cancer cell types, including leukemia, liver, colon, prostate, pancreas, bone, or brain cancer [9–16]. The molecular mechanism mediating these antitumoral effects is still under debate. Several modes of action, including interference with Wnt signaling, inhibition or induction of autophagy, destruction of the cytoskeleton, or destabilization of mitochondrial membrane potential, have been described [12–14, 17, 18]. We have demonstrated before in human CC cells that Sal is able to overcome apoptosis resistance, and affects tumor cell proliferation and migration [19]. The aim of this study was to investigate further molecular mechanisms of Sal by analyzing autophagic flux in human CC cells after exposure to Sal, and to analyze the therapeutic effectiveness of Sal for the treatment of CC in mouse models *in vivo*.

## RESULTS

### Salinomycin exposure results in impaired tumor cell proliferation

**Figure 1:**
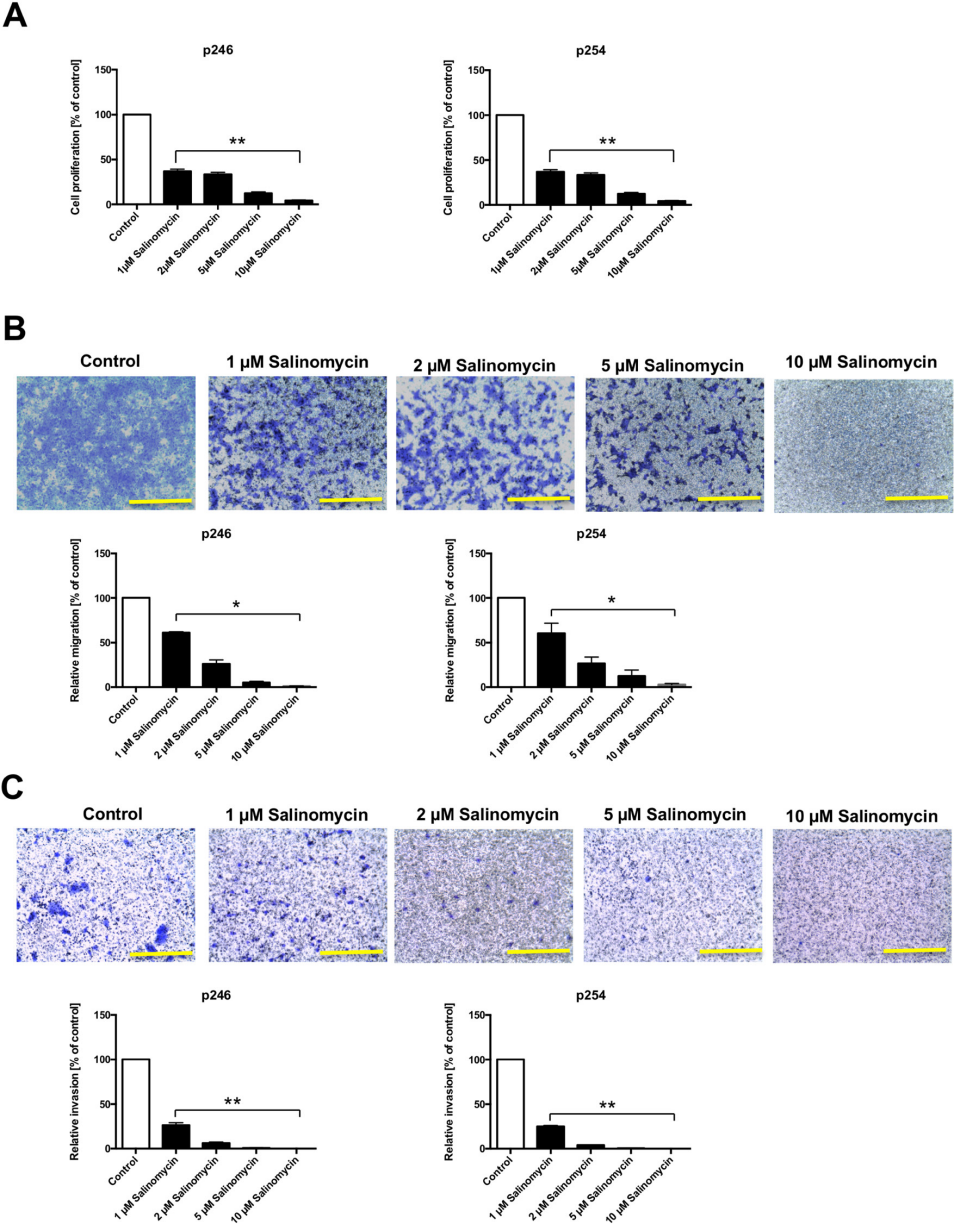
Reduced murine tumor cell proliferation and deceased migration and invasion after treatment with Salinomycin (**A**) For the assessment of tumor cell proliferation after exposure to Sal, 5 × 10^3^ p246 and p254 cells were cultured in microtiter plates in the absence or presence of increasing concentrations of Salinomycin (1, 2, 5, and 10 µM). Treatment was performed for 48 h. Results are displayed as a summary of at least three independent experiments as mean ± SD; ^**^P < 0.001 compared with control. (**B**) For transwell analysis of tumor cell migration, 1 × 10^5^ p246 or p254 cells were seeded in six-well plates equipped with a transwell insert and treated with increasing concentrations of Salinomycin (1, 2, 5, and 10 µM). After 48 h, the medium was changed and the cells were further incubated for another 48 h. Afterward, the membranes were stained with crystal violet solution, and the migrated cells were isolated from the lower side of the membrane and quantified by ELISA reader. (**C**) Alternatively, p246 and p254 cells were cultured in Matrigel-coated transwell inserts. After 48 h of treatment and 48 h of further incubation, the number of invasive migrated cells was quantified as described previously. Results are shown as representative images of stained membranes at a magnification of 100 or as summary or at least three independent experiments as mean ± SD; ^*^P < 0.05, ^**^P < 0.001 compared with control. Scale bars = 100 µm.

To analyze the effect of Sal on murine CC cells, p246 and p254 cells were exposed to increasing concentrations of Sal (1, 2, 5, and 10 µM) for 48 h. As demonstrated in Figure 1A, Sal treatment significantly reduced tumor cell proliferation dose-dependently in both p246 and p254 cells after 48 h of treatment. Similar results were obtained when cells were treated for 24 or 72 h (data not shown).

### Salinomycin reduces tumor cell migration and invasion

After demonstrating the antiproliferative effect of Sal on murine CC cells, we analyzed the effect of Sal on tumor cell migration and invasion, applying a transwell assay. As demonstrated in Figure 1B, treatment with Sal significantly decreased transmembrane migration of p246 and p254 cells after treatment for 48 h and further incubation for another 48 h. Murine CC cell invasion through an artificial extracellular matrix using Matrigel-coated membranes was likewise significantly impaired in response to Sal administration in a dose-dependent manner (Figure 1C).

### Salinomycin induces apoptosis in murine CC cells

**Figure 2:**
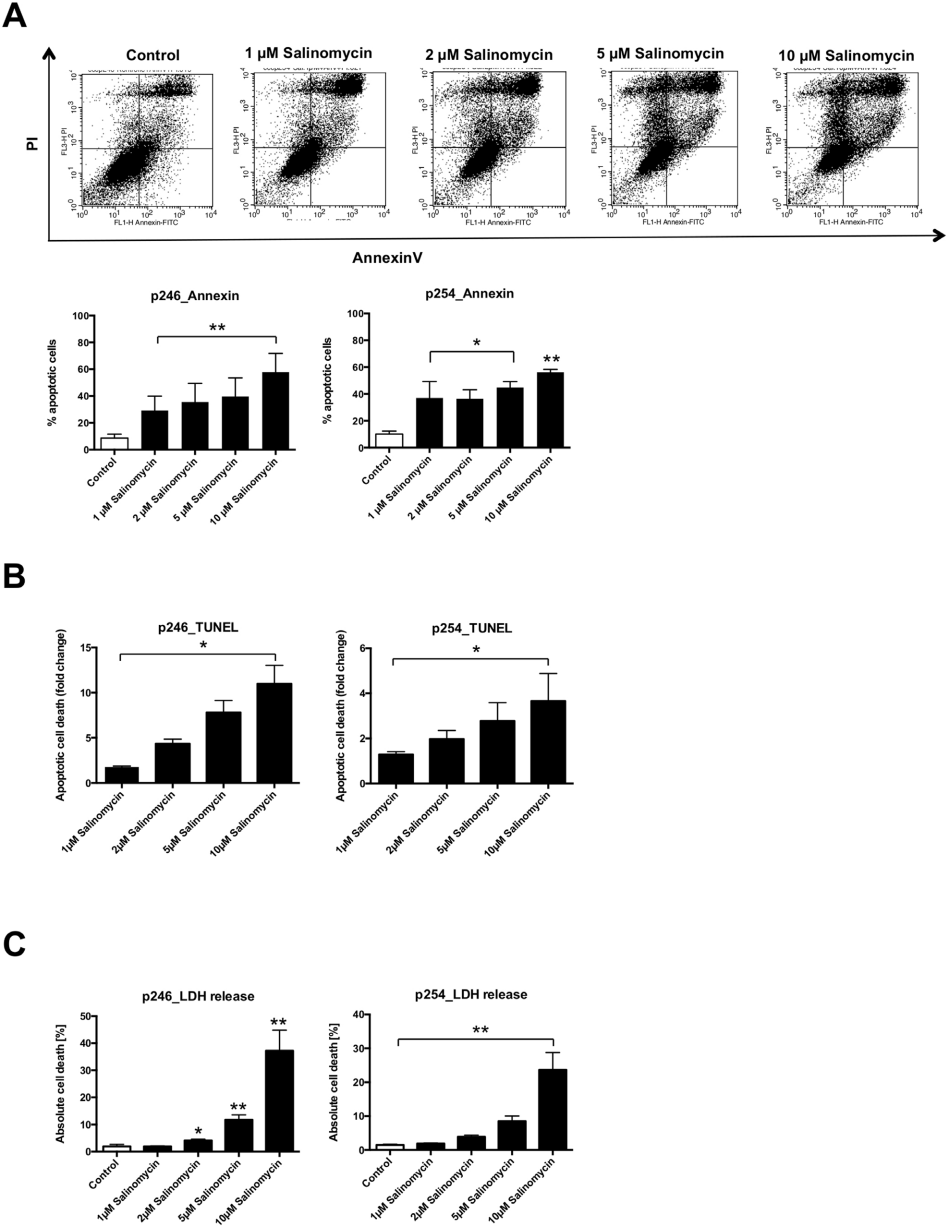
Treatment with Salinomycin induces apoptosis in murine CC cells (**A**) A total of 0.5 × 10^6^ p246 or p254 cells were seeded in six-well plates and grown until confluence following exposure to increasing concentrations of Salinomycin (1, 2, 5, and 10 µM) for 24 h. Detection of apoptosis was performed using AnnexinV-FITC and propidium iodide staining, and cells were analyzed by flow cytometry. Cell death was further determined by quantification of DNA fragmentation (**B**) and LDH release assay (**C**). Results are displayed as representative dot blots or as a summary of at least three independent experiments; ^*^P < 0.05; ^**^P < 0.001 compared with control.

Next, we analyzed whether Sal induces apoptosis in p246 and p254 cells. As demonstrated in Figure 2A, Sal treatment was associated with an increased amount of apoptotic (AnnexinV/propidium iodide–positive) murine CC cells. This effect was dose-dependent in both p246 and p254 cells (Figure 2A). Exposure to Sal likewise resulted in increased DNA fragmentation in p246 and p254 cells, respectively (Figure 2B). Cell death was further confirmed in a lactate dehydrogenase (LDH) release assay. As demonstrated in Figure 2C, treatment with Sal resulted in a dose-dependent increase of LDH release in murine tumor cells (Figure 2C) after exposure to Sal for 24 h.

### Salinomycin treatment inhibits murine CC growth *in vivo*

**Figure 3:**
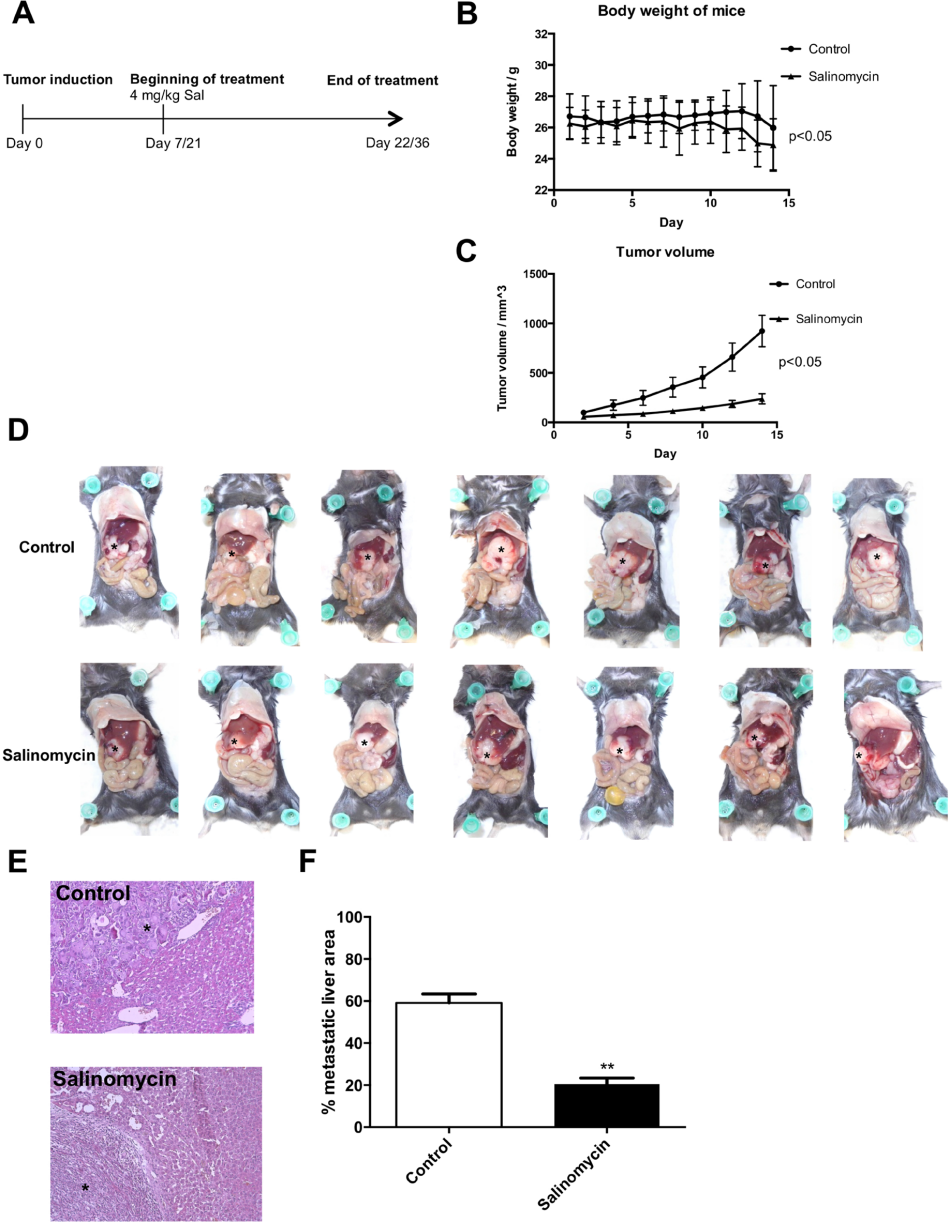
Salinomycin inhibits murine cholangiocarcinoma growth *in vivo* (**A**) Subcutaneous cholangiocarcinoma growth in Bl/6-mice was induced through injection of p254 cells into the flank of the animals. Induction of intrahepatic tumor growth was induced through electroporation of sleeping beauty–based oncogenic transposon plasmids into the left liver lobe, and resulted in KRas/Akt2-activation and p53-knockout in hepatocytes. After tumor growth, treatment ensued with either vehicle or Salinomycin. (**B**) Effect of Salinomycin treatment on body weight (g) of mice. (**C**) After 2 weeks of treatment, Salinomycin significantly inhibited colorectal cancer growth in the subcutaneous tumor model. Results are shown as mean tumor volume ± SD. (**D**–**F**) Distribution of intrahepatic cholangiocarcinoma spread in the liver of mice (indicated as^*^) was also markedly reduced after treatment with Salinomycin. Results are shown as representative images of cholangiocarcinoma liver spread, H&E stained sections, or mean of the percentage of metastatic area ± SD of seven individual experiments; ^**^P < 0.001 compared with control.

Two independent tumor models (subcutaneous and intrahepatic) were applied to investigate the effect of Sal on murine CC growth *in vivo*. Seven animals were treated in each group with vehicle or Sal for 2 weeks (Figure 3A). Chemotherapy was well-tolerated, as documented by the general health status of the animals and body weight during the treatment (Figure 3B).

In the subcutaneous tumor model, Sal effectively inhibited CC growth after 14 days of daily treatment compared with vehicle-treated animals (Figure 3C). This effect was statistically significant and already visible after 5 days of treatment. Although tumors in untreated animals rapidly expanded, an increase in tumor volume was inhibited upon Sal treatment. To reflect a metastatic setting of nontransplant, autochthonously grown intrahepatic CC model, we used the transposon-based intrahepatic CC induction. Therefore, local transfection of the liver parenchyma by electroporation with transposon DNA resulted in transposase-mediated oncogenic KRas-G12V-insertion and Akt2-activation combined with Cre-mediated p53-knockout. This procedure resulted in the formation of an intrahepatic tumor with distant metastases. Sal likewise inhibited intrahepatic CC growth. In addition to reduced size of the primary tumor within the liver of the mice, metastatic liver spread was markedly reduced in Sal-treated animals compared with animals treated with corn oil (control group, Figure 3D–3F).

### Salinomycin inhibits autophagic flux in human CC cells

**Figure 4:**
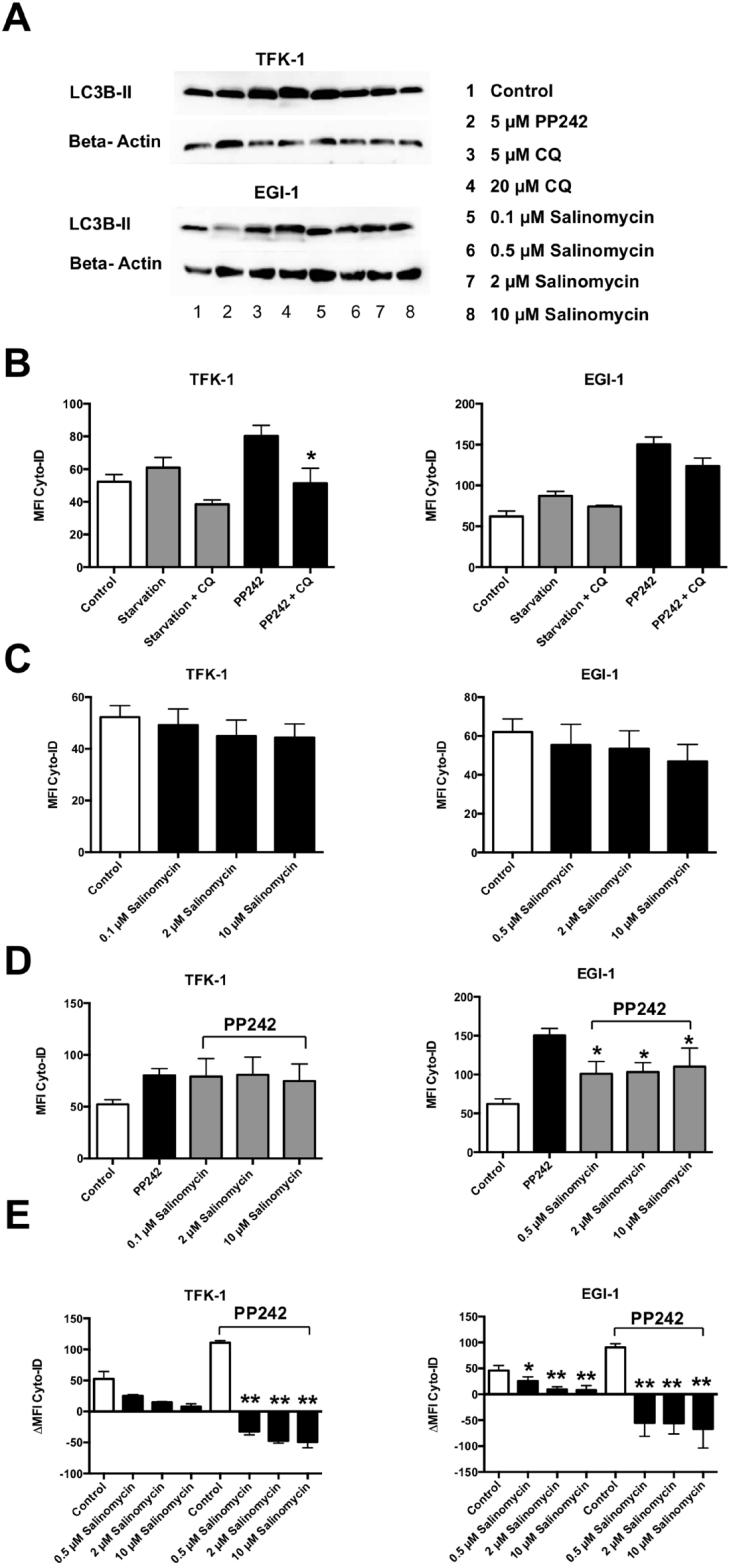
Salinomycin inhibits autophagic flux in human CC cells TFK-1 and EGI-1 cells were exposed to pharmacological activators (PP242) or inhibitors (ACH, CQ) and increasing concentrations of Salinomycin (0.1, 0.5, 2, and 10 µM) and analyzed for activity of autophagy. (**A**) Western blot analysis of LC3-II in TFK-1 (up) and EGI-1 cells indicates Salinomycin-induced accumulation of LC3-II as a result of inhibition of autophagy. (**B**) Basic and activated autophagic flux in TFK-1 and EGI-1 cells was analyzed by measurement of autophagic compartments. Basic and activated autophagic flux was counteracted by the addition of CQ. (**C**) Basic activation of autophagy after exposure to Salinomycin for 24 h. (**D**) PP242-activated autophagic flux was partially counteracted by Sal. (**E**) Treatment with Salinomycin counteracts PP242-induced activation of autophagy and is associated with decreased accumulation of autophagic compartments after blockage of autophagolysosomal degradation, indicating inhibition of autophagic flux in TFK-1 and EGI-1 cells. Results are displayed as a summary of at least three independent experiments as mean ± SD; ^*^P < 0.05 compared with control.

To assess the effect of Sal on autophagy in human CC cells, we treated TFK-1 and EGI-1 cells for 24 h with pharmacological activators (PP242) or inhibitors of autophagy (chloroquine (CQ)), and increasing concentrations of Sal (0.1, 0.5, 2, and 10 µM). Semiquantitative western blot analysis demonstrated a Sal-induced increase of LC3-II (Figure 4A). Accumulation of LC3-II was also detected, as expected, after CQ-mediated blockage of autophagolysosomal degradation.

Next, we evaluated autophagic activity in human CC cells. Activation of autophagy by starvation of exposure to PP242 resulted in an increased amount of autophagic compartments in both TFK-1 and EGI-1 cells, which was counteracted by the addition of CQ (Figure 4B). Basic analysis of autophagic flux after treatment with increasing concentrations of Sal resulted in a moderate decrease of autophagic flux in human CC cells (Figure 4C). Activation of autophagy in TFK-1 and EGI-1 cells after PP242-incubation was in part counteracted by Sal (Figure 4D). Those effects were more visible in EGI-1 cells compared with TFK-1 cells. To elucidate the precise effect of Sal on autophagic activity in human CC cells, we analyzed the accumulation of autophagic compartments in PP242-activated conditions after the blockade of autophagolysosomal degradation by CQ. For this, TFK-1 and EGI-1 cells were exposed to increasing concentrations of Sal (0.5, 2, and 10 µM) for 24 h and compared with untreated cells. Sal reduced autophagic flux in TFK-1 and EGI-1 cells in a dose-dependent manner. This was also observed when autophagy was activated by PP242 (Figure 4E). Similar results were obtained when autophagolysosomal degradation was induced by ACH (Supplementary Figure 1).

### Exposure to Salinomycin results in dysfunctional mitochondria and increased production of reactive oxygen species

**Figure 5:**
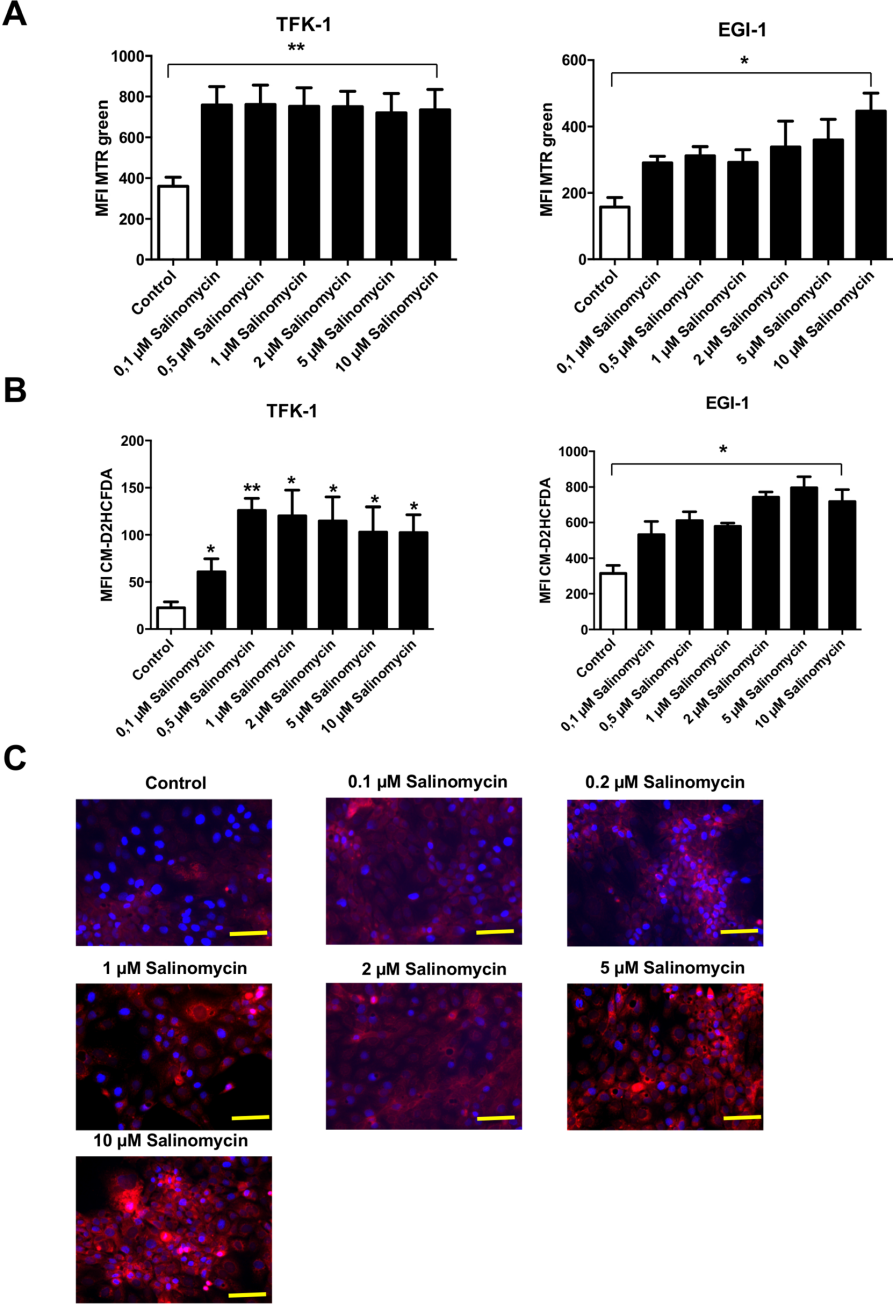
Treatment with Salinomycin results in accumulation of mitochondrial mass and increased generation of ROS (**A**) Analysis of total mitochondrial mass in TFK-1 and EGI-1 cells after exposure to increasing concentrations of Salinomycin (0.1, 0.5, 2, 5, and 10 μM) was assessed using MTR green. After 24 h, accumulation of mitochondrial mass was observed in both TFK-1 and EGI-1 cells. (**B**) Increased generation of ROS after treatment with Salinomycin in TFK-1 and EGI-1 cells using CM-H2DCFDA staining and analyzed by flow cytometry. (**C**) Immunostaining of TFK-1 cells after exposure of Sal confirmed increased ROS generation. Results are displayed as a summary of at least three independent experiments as mean ± SD or as representative image capture by fluorescence microscopy; ^*^P < 0.05 compared with control. Scale bars = 50 µm.

Based on the observation that inhibition of autophagy is associated with an increased amount of dysfunctional mitochondria (and consequently increased production of reactive oxygen species (ROS) [12]), we analyzed whether treatment with Sal results in accumulation of mitochondrial mass. As demonstrated in Figure 5A, exposure to Sal for 24 h results in an increased amount of mitochondrial mass in both TFK-1 and EGI-1 cells. It is remarkable that in TFK-1 cells, an increase of mitochondrial mass was already observed after exposure to low concentrations of Sal. In EGI-1 cells, treatment with Sal resulted in a dose-dependent accumulation of mitochondrial mass (Figure 5A). Given that accumulation of mitochondrial mass is an indicator of mitochondrial dysfunction, we analyzed the generation of ROS after exposure to Sal. Treatment with Sal for 24 h led to increased ROS generation in TFK-1 and EGI-1 cells, even after exposure to low concentrations of the agent (Figure 5B, 5C and Supplementary Figure 2).

## DISCUSSION

Our study provides evidence that Sal exerts its anticancer effects in CC *in vivo* by inhibition of subcutaneous and intrahepatic tumor growth. Furthermore, we are able to demonstrate that exposure of human CC cells to Sal is accompanied by inhibition of autophagic flux, leading to accumulation of dysfunctional mitochondria and increased production of ROS.

CC is the second-most common primary liver tumor, and is associated with poor prognosis—especially as a result of irresectability in advanced stage of disease and ineffective chemotherapy [20]. Therefore, innovative and effective therapies are of utmost importance. We have demonstrated previously that Sal overcomes apoptosis resistance of human CC cells *in vitro* [19].

To further evaluate the potential use of Sal for the treatment of CC, we established a murine model to investigate the effectiveness of Sal in CC *in vivo*. First, we were able to demonstrate that Sal exerts its pro-apoptotic effect in two murine CC cell lines in a dose-dependent manner, accompanied by inhibition of tumor cell migration and invasion. Next, we induced subcutaneous tumor growth in C57-BL/6-mice and observed statistically significant inhibition of tumor growth compared with vehicle-treated animals. To mimic the clinical situation of CC, intrahepatic tumor growth was initiated via electroporation of oncogenic transposon plasmids. This resulted in KRas-activation and p53-knockout in hepatocytes, leading to the development of intrahepatic CC growth [21]. Again, Sal inhibits CC growth and intrahepatic tumor spread *in vivo*. These findings demonstrate for the first time the effectiveness of Sal in a clinically relevant CC model. Additionally, inhibition of intrahepatic tumor spread has an important clinical impact, given that common chemotherapies have to be regarded rather in a palliative context [6].

Next, we aimed to understand the underlying molecular mechanism of Sal, which causes cell death in human CC cells. Based on our observation that Sal induces apoptosis in human CC cells [19], we hypothesized that interference with the activity of autophagy is related to the effectiveness of Sal. Because of the observation that tumor cells experience more inherent stress than benign cells, in part because of treatment with chemotherapy, they are regarded as dependent on functional autophagic activity [22]. Having demonstrated before that Sal inhibits autophagic flux in human hepatocellular carcinoma cells [12], we hypothesized that inhibition of autophagic activity is a possible underlying molecular mechanism for the effectiveness of Sal in hepatobiliary malignancies. Supported by the following findings, we conclude that Sal-mediated inhibition of autophagic flux contributes to the effectiveness of the drug in CC. First, we demonstrated that Sal acts as an inhibitor of basic and pharmacologically induced autophagic flux in human CC cells. These inhibitory effects are more pronounced than the effects mediated by established inhibitors of autophagy. Second, inhibition of autophagic flux is associated with accumulation of dysfunctional mitochondria and increased generation of ROS. It has been reported before that interference-free autophagy is crucial for the removal of dysfunctional mitochondria [23, 24], which are necessary for the elimination of accumulated ROS. Reactive oxygen species–mediated induction of apoptosis by Sal has been reported before in other types of cancer [10, 12, 16, 25]. This let us suppose that Sal exerts its anticancer effects via generation of ROS via different molecular pathways.

The role of Sal on autophagy in cancer cells is still under debate. There are numerous reports indicating that Sal inhibits autophagy and, consequently, impairs tumor cell survival [10, 12, 26–29]. Besides our own observations in HCC cells [12], two studies demonstrated induction of apoptosis in breast cancer cells after inhibition of autophagic flux by Sal [28, 29]. In contrast, other studies propose that Sal acts as an activator of autophagy, leading to apoptotic cell death [10, 18, 26, 27]. Similar to our observations, Sal-mediated cell death via induction of autophagy is associated with impaired mitochondrial function and increased production of ROS. Those paradoxical observations regarding the effect of Sal on the activity of autophagy might be explained by the assumption that the molecular mechanism differs between different types of cancer. All in common is that Sal-mediated interference of autophagic flux results in tumor cell death.

## CONCLUSIONS

Taken together, our data demonstrate that Sal is able to effectively inhibit CC growth in two different tumor models *in vivo*. Furthermore, we provide evidence that Sal suppresses autophagic flux in human CC cells, representing a possible underlying molecular mechanism. Further studies are necessary to compare the effectiveness of Sal and common chemotherapy for CC *in vivo*. This would help to delineate the potential clinical use of Sal for patients with CC.

## MATERIALS AND METHODS

### Cell lines and culture

The murine CC cell lines p246 and p254 were obtained from murine models of intrahepatic CC [21], and were used to analyze the effect of Sal *in vitro*. Cells were cultured in RPMI medium + Glutamax (Invitrogen, Darmstadt, Germany) supplemented with 10% fetal calf serum, penicillin (50 U/mL), and streptomycin (50 mg/L) at 37°C and 5% CO_2_. The two human CC cell lines EGI-1 and TFK-1 were used to analyze the effect of Sal on autophagic flux. The EGI-1 and TFK-1 cells were cultured in culture medium (RPMI 1640 + Glutamax, supplemented with 10% fetal bovine serum, 10 mM HEPES-Buffer, 1% MEM nonessential amino acids, penicillin (50 U/mL), and streptomycin (50 mg/L)) (Invitrogen) at 37°C and 5% CO_2_. High balanced salt solution containing 6 mM glucose was used as starvation medium.

### Chemicals

Sal was purchased from Sigma-Aldrich (St. Louis, MO, USA) and was dissolved in dimethyl sulfoxide for *in vitro* analysis or in corn oil for *in vivo* applications. The established inhibitors or activators of autophagy chloroquine (CQ), ammonium chloride (ACH), and PP242 were obtained from Enzo Life Sciences [30]. Stock solutions were stored at 20°C. Antibodies for β-actin light chain protein 3 (LC3) were purchased from Sigma Aldrich.

### Proliferation

A total of 5 × 10^3^ murine CC cells were cultured in 96-well flat-bottom plates. Cells were exposed to increasing concentrations of Sal (1, 2, 5, and 10 µM) for 48 h. Cell proliferation was measured using the WST-1 assay, which is based on the cleavage of the tetrazolium salt WST-1 into formazan by healthy cells. Formazan formation was quantified by measuring the absorbance at 450 nm according to the manufacturer’s instructions.

### Tumor cell migration and invasion

Tumor cell migration was investigated using transwell chambers (Cell Biolabs, San Diego, CA, USA) equipped with an 8-µm pore polycarbonate membrane as described in [31]. A total of 1 × 10^5^ murine CC cells were seeded in the upper compartment of the membrane in culture medium without fetal calf serum. The lower compartment of the chamber was filled with culture medium containing 20% fetal calf serum. Cells were cultured in the absence or presence of Sal for 48 h. Afterward, the medium was changed and the cells were incubated for another 48 h. Membranes were stained with crystal violet solution, and the migrated cells on the lower side of the membrane were isolated from the membrane and quantified by measurement of the absorbance at 540 nm according to the manufacturer’s instructions and as described in [11].

Tumor cell invasiveness was analyzed by seeding 1 × 10^5^ cells in Matrigel-coated membranes of transwell chambers (Cell Biolabs) according to the manufacturer’s instructions and as described in [32]. Tumor cell invasion assay was further performed as described previously for tumor cell migration assay.

### Apoptosis

Cells were analyzed for apoptosis induction after exposure to Sal for 24 h using the AnnexinV apoptosis detection kit (BD Biosciences, San Jose, CA, USA), according to the manufacturer’s instructions as described in [12, 19]. Apoptosis was further quantified by quantification of DNA fragmentation in cultured p254 and p246 cells using the HT Titer TACS Assay Kit (Trevigen, Gaithersburg, MD, USA) according to the manufacturer’s instructions and as described in [33]. Tumor cell death was analyzed applying the LDH Cytotoxicity Assay Kit (Thermo Fisher Scientific, Waltham, MA, USA) following the manufacturer’s protocol and as described in [34].

### Animal models and treatment

Animal experiments were carried out in 6- to 10-week-old C57-BL/6-mice purchased from Charles River Laboratories. Animals were housed under standard conditions with free access to food and water under constant environmental conditions with a 12-h day–night cycle. Isoflurane was used for inhalation anesthesia. For the subcutaneous CC model, 1 × 10^6^ p254 cells were injected in 50-µL Matrigel (BD Biosciences) into the flank of mice. After 7 days and a tumor volume of approximately 50 mm^3^, the animals were randomized and treated either with corn oil (control group) or Sal (4 mg/kg). Tumor volume was assessed daily during chemotherapy for a total duration of 14 days. The effect of Sal on intrahepatic tumor growth is based on a transposon-mediated, autochthonously grown cholangiocarcinoma model, as described in [21]. In brief, electroporation of sleeping beauty–based oncogenic transposon plasmids into the left liver lobe of 6- to 8-week-old p53fl/fl mice (Strain B6129P2-Trp53^tm1Brn^/J) resulted in KRas-activation and p53-knockout in hepatocytes, leading to the development of intrahepatic CC growth [21]. After tumor induction within 3 weeks, the animals were treated with Sal (4 mg/kg) for 14 days. Afterward, intrahepatic tumor growth and spread were analyzed based on hematoxylin and eosin (H&E) staining, as described in [11]. The metastatic area within the livers was determined morphometrically by applying ImageJ software. Twenty pictures from each H&E stained slide (10 slides per animal) were randomly taken, and the metastatic lesions marked. Pixels within the marked areas were related to the overall pixel count by Image J software, and the mean ± standard deviation (SD) was calculated and expressed as a percentage of metastatic area in correlation to the whole liver.

The local animal care committees of the University of Heidelberg and Hannover Medical School approved all experiments.

### Analysis of Sal-mediated effect on CC cell autophagic activity

Autophagic flux in human CC cell lines was analyzed by conversion of LC3-I to LC3-II and determined by western blotting using an anti-LC-3B antibody (Sigma-Aldrich) as described in [12]. In brief, after drug treatment, nuclear protein was isolated (Life Technologies, Carlsbad, CA, USA). Protein content was determined by applying the BCA Protein Assay Kit (Life Technologies). Equal amounts of protein were separated by 10% SDS-PAGE and transferred to polyvinylidene difluoride membranes (Merck Millipore, Billerica, MA, USA). The membranes were incubated overnight with primary antibodies against human LC3-II. After washing, membranes were incubated and developed with a horseradish peroxidase-conjugated secondary antibody (Life Technologies). β-actin served as an internal control.

Autophagic flux also was determined using the Cyto-ID Autophagy Detection Kit (Enzo Life Sciences, Farmingdale, NY, USA), according to the manufacturer’s instructions described in [12]. This assay is based on a specific dye that selectively stains autophagic compartments. Autophagic flux is therefore quantified as accumulation of autophagic compartments in basic or activated conditions (starvation medium or PP242-incubation) after the blockage of autophagolysosomal degradation by CQ or ACH. Autophagic flux is calculated by subtracting the Cyto-ID mean fluorescence intensity (MFI) of the sample without CQ/ACH from the Cyto-ID MFI of the sample with CQ/ACH for each condition using the following formula: ΔMFI Cyto-ID = MFI Cyto-ID (+CQ/ACH) MFI Cyto-ID (-CQ/ACH).

### Measurement of mitochondrial mass and ROS generation

Mitochondrial mass was determined by flow cytometry using MitoTracker Green FM (MTR green) staining (Molecular Probes, Eugene, OR, USA), according to the manufacturer’s instructions and described in [12]. The ROS generation was determined by flow cytometry, applying 5-(and-6)-chloromethyl-2′,7′-dichlorodihydrofluorescein diacetate, acetyl ester (CM-H2DCFDA) staining (Invitrogen) according to the manufacturer’s instructions and described in [12]. The ROS generation was further analyzed by applying the MitoTracker Probes Red Fm Kit (Invitrogen) according to the manufacturer’s instructions and described in [35].

### Immunohistochemistry

Paraffin fixed-tissue samples were cut into sections of 5 µm, and routine H&E staining was performed to evaluate histomorphological features.

### Statistical analysis

Statistical analysis was performed using GraphPad Prism 6 (GraphPad Software, La Jolla, CA, USA). Student’s t-test or analysis of variance was applied as appropriate. Differences were regarded as statistically significant with *P* < 0.05. Results were expressed as mean ± SD of at least three independent experiments.

## SUPPLEMENTARY MATERIALS FIGURES



## Abbreviations

ACH: ammonium chloride
CC: cholangiocarcinoma
CQ: chloroquine
ROS: reactive oxygen species
Sal: Salinomycin

## References

[1] Lazaridis KN, Gores GJ (2005). Cholangiocarcinoma. Gastroenterology.

[2] LaFemina J, Jarnagin WR (2012). Surgical management of proximal bile duct cancers. Langenbecks Arch Surg.

[3] Neuhaus P, Thelen A, Jonas S, Puhl G, Denecke T, Veltzke-Schlieker W, Seehofer D (2012). Oncological superiority of hilar en bloc resection for the treatment of hilar cholangiocarcinoma. Ann Surg Oncol.

[4] Rea DJ, Rosen CB, Nagorney DM, Heimbach JK, Gores GJ (2009). Transplantation for cholangiocarcinoma: when and for whom?. Surg Oncol Clin N Am.

[5] Valle J, Wasan H, Palmer DH, Cunningham D, Anthoney A, Maraveyas A, Madhusudan S, Iveson T, Hughes S, Pereira SP, Roughton M, Bridgewater J, ABC-02 Trial Investigators (2010). Cisplatin plus gemcitabine versus gemcitabine for biliary tract cancer. N Engl J Med.

[6] Valle JW, Furuse J, Jitlal M, Beare S, Mizuno N, Wasan H, Bridgewater J, Okusaka T (2014). Cisplatin and gemcitabine for advanced biliary tract cancer: a meta-analysis of two randomised trials. Ann Oncol.

[7] Naujokat C, Fuchs D, Opelz G (2010). Salinomycin in cancer: A new mission for an old agent. Mol Med Rep.

[8] Gupta PB, Onder TT, Jiang G, Tao K, Kuperwasser C, Weinberg RA, Lander ES (2009). Identification of selective inhibitors of cancer stem cells by high-throughput screening. Cell.

[9] Ketola K, Hilvo M, Hyötyläinen T, Vuoristo A, Ruskeepää AL, Orešič M, Kallioniemi O, Iljin K (2012). Salinomycin inhibits prostate cancer growth and migration via induction of oxidative stress. Br J Cancer.

[10] Kim SH, Choi YJ, Kim KY, Yu SN, Seo YK, Chun SS, Noh KT, Suh JT, Ahn SC (2016). Salinomycin simultaneously induces apoptosis and autophagy through generation of reactive oxygen species in osteosarcoma U2OS cells. Biochem Biophys Res Commun.

[11] Klose J, Eissele J, Volz C, Schmitt S, Ritter A, Ying S, Schmidt T, Heger U, Schneider M, Ulrich A (2016). Salinomycin inhibits metastatic colorectal cancer growth and interferes with Wnt/β-catenin signaling in CD133+ human colorectal cancer cells. BMC Cancer.

[12] Klose J, Stankov MV, Kleine M, Ramackers W, Panayotova-Dimitrova D, Jäger MD, Klempnauer J, Winkler M, Bektas H, Behrens GM, Vondran FW (2014). Inhibition of autophagic flux by salinomycin results in anti-cancer effect in hepatocellular carcinoma cells. PLoS One.

[13] Lu D, Choi MY, Yu J, Castro JE, Kipps TJ, Carson DA (2011). Salinomycin inhibits Wnt signaling and selectively induces apoptosis in chronic lymphocytic leukemia cells. Proc Natl Acad Sci USA.

[14] Schenk M, Aykut B, Teske C, Giese NA, Weitz J, Welsch T (2015). Salinomycin inhibits growth of pancreatic cancer and cancer cell migration by disruption of actin stress fiber integrity. Cancer Lett.

[15] Wang F, Dai W, Wang Y, Shen M, Chen K, Cheng P, Zhang Y, Wang C, Li J, Zheng Y, Lu J, Yang J, Zhu R (2014). The synergistic *in vitro* and *in vivo* antitumor effect of combination therapy with salinomycin and 5-fluorouracil against hepatocellular carcinoma. PLoS One.

[16] Xipell E, Gonzalez-Huarriz M, Martinez de Irujo JJ, García-Garzón A, Lang FF, Jiang H, Fueyo J, Gomez-Manzano C, Alonso MM (2016). Salinomycin induced ROS results in abortive autophagy and leads to regulated necrosis in glioblastoma. Oncotarget.

[17] Managò A, Leanza L, Carraretto L, Sassi N, Grancara S, Quintana-Cabrera R, Trimarco V, Toninello A, Scorrano L, Trentin L, Semenzato G, Gulbins E, Zoratti M, Szabò I (2015). Early effects of the antineoplastic agent salinomycin on mitochondrial function. Cell Death Dis.

[18] Verdoodt B, Vogt M, Schmitz I, Liffers ST, Tannapfel A, Mirmohammadsadegh A (2012). Salinomycin induces autophagy in colon and breast cancer cells with concomitant generation of reactive oxygen species. PLoS One.

[19] Lieke T, Ramackers W, Bergmann S, Klempnauer J, Winkler M, Klose J (2012). Impact of Salinomycin on human cholangiocarcinoma: induction of apoptosis and impairment of tumor cell proliferation *in vitro*. BMC Cancer.

[20] Ghouri YA, Mian I, Blechacz B (2015). Cancer review: cholangiocarcinoma. J Carcinog.

[21] Gürlevik E, Fleischmann-Mundt B, Armbrecht N, Longerich T, Woller N, Kloos A, Hoffmann D, Schambach A, Wirth TC, Manns MP, Zender L, Kubicka S, Kühnel F (2013). Adjuvant gemcitabine therapy improves survival in a locally induced, R0-resectable model of metastatic intrahepatic cholangiocarcinoma. Hepatology.

[22] Mathew R, Karantza-Wadsworth V, White E (2007). Role of autophagy in cancer. Nat Rev Cancer.

[23] Kanki T, Klionsky DJ (2010). The molecular mechanism of mitochondria autophagy in yeast. Mol Microbiol.

[24] Kim I, Rodriguez-Enriquez S, Lemasters JJ (2007). Selective degradation of mitochondria by mitophagy. Arch Biochem Biophys.

[25] Kim KY, Park KI, Kim SH, Yu SN, Lee D, Kim YW, Noh KT, Ma JY, Seo YK, Ahn SC (2017). Salinomycin Induces Reactive Oxygen Species and Apoptosis in Aggressive Breast Cancer Cells as Mediated with Regulation of Autophagy. Anticancer Res.

[26] Jangamreddy JR, Ghavami S, Grabarek J, Kratz G, Wiechec E, Fredriksson BA, Rao Pariti RK, Cieślar-Pobuda A, Panigrahi S, Łos MJ (2013). Salinomycin induces activation of autophagy, mitophagy and affects mitochondrial polarity: differences between primary and cancer cells. Biochim Biophys Acta.

[27] Jangamreddy JR, Panigrahi S, Łos MJ (2015). Monitoring of autophagy is complicated—salinomycin as an example. Biochim Biophys Acta.

[28] Pellegrini P, Dyczynski M, Sbrana FV, Karlgren M, Buoncervello M, Hägg-Olofsson M, Ma R, Hartman J, Bajalica-Lagercrantz S, Grander D, Kharaziha P, De Milito A (2016). Tumor acidosis enhances cytotoxic effects and autophagy inhibition by salinomycin on cancer cell lines and cancer stem cells. Oncotarget.

[29] Yue W, Hamaï A, Tonelli G, Bauvy C, Nicolas V, Tharinger H, Codogno P, Mehrpour M (2013). Inhibition of the autophagic flux by salinomycin in breast cancer stem-like/progenitor cells interferes with their maintenance. Autophagy.

[30] Mizushima N, Yoshimori T, Levine B (2010). Methods in mammalian autophagy research. Cell.

[31] Boyden S (1962). The chemotactic effect of mixtures of antibody and antigen on polymorphonuclear leucocytes. J Exp Med.

[32] Repesh LA (1989). A new *in vitro* assay for quantitating tumor cell invasion. Invasion Metastasis.

[33] Sokalska A, Wong DH, Cress A, Piotrowski PC, Rzepczynska I, Villanueva J, Duleba AJ (2010). Simvastatin induces apoptosis and alters cytoskeleton in endometrial stromal cells. J Clin Endocrinol Metab.

[34] Grubor-Bauk B, Yu W, Wijesundara D, Gummow J, Garrod T, Brennan AJ, Voskoboinik I, Gowans EJ (2016). Intradermal delivery of DNA encoding HCV NS3 and perforin elicits robust cell-mediated immunity in mice and pigs. Gene Ther.

[35] Kuznetsov AV, Kehrer I, Kozlov AV, Haller M, Redl H, Hermann M, Grimm M, Troppmair J (2011). Mitochondrial ROS production under cellular stress: comparison of different detection methods. Anal Bioanal Chem.

