# Supplementary material for: Salinomycin inhibits cholangiocarcinoma growth by inhibition of autophagic flux
Source: Oncotarget. 2017 Dec 16;9(3):3619–30. doi: 10.18632/oncotarget.23339 (PMC5790487; doi:10.18632/oncotarget.23339)
Supplement: Supplementary file 1 [file oncotarget-09-3619-s001.pdf]

Salinomycin inhibits cholangiocarcinoma growth by inhibition of autophagic flux

SUPPLEMENTARY MATERIALS

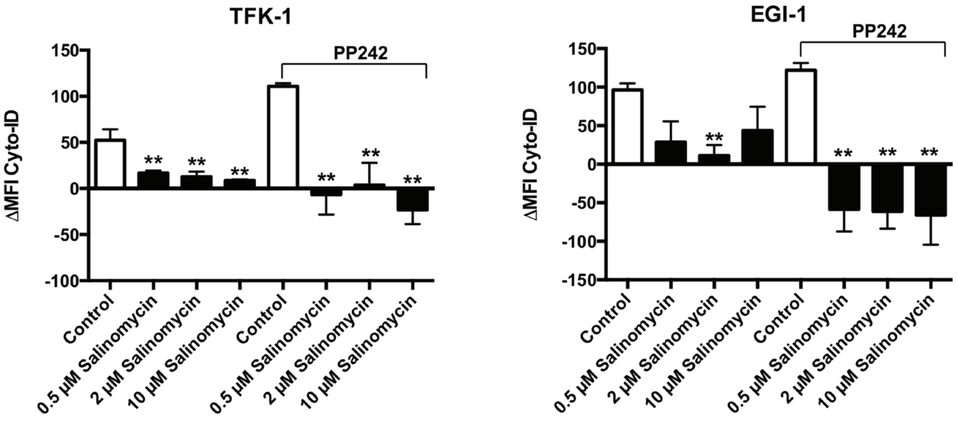

Supplementary Figure 1: Salinomycin inhibits autophagic flux in human CC cells. Treatment with Salinomycin counteracts PP242-induced activation of autophagy and is associated with decreased accumulation of autophagic compartments after blockage of autophagolysosomal degradation by ACH, indicating inhibition of autophagic flux in TFK-1 and EGI-1 cells. TFK-1 (**A**) and EGI-1 cells (**B**) were treated with increasing concentrations of Salinomycin (0.5, 2, and 10 µM for 24 h). The generation of autophagic compartments was analyzed in basic and PP242-activated conditions with or without the addition of ACH. Results are displayed as a summary of at least three independent experiments as mean ± SD; $^{*}P < 0.05$ compared with control.

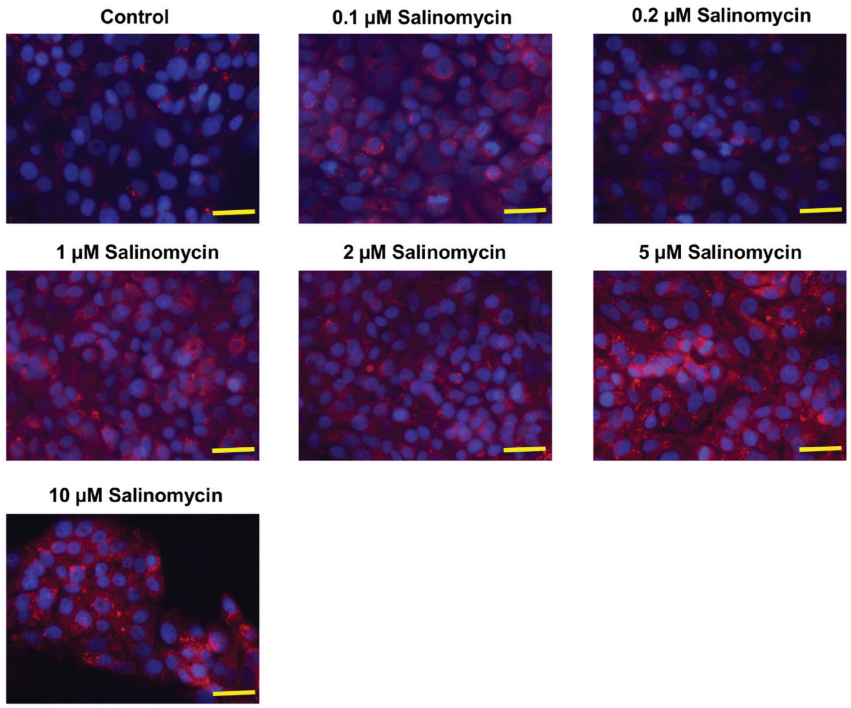

Supplementary Figure 2: Salinomycin increases ROS generation in EGI-1 cells. The EGI-1 cells were exposed to increasing concentrations of Salinomycin (0.1, 0.5, 1, 2, 5, and 10 µM) for 24 h. Immunostaining for ROS revealed a dose-dependent increase in ROS generation after exposure to Sal. Results are displayed as representative image capture by fluorescence microscopy. Scale bars = 50 µM.
